# Evaluation of the influence of platelet-rich plasma (PRP), platelet lysate (PL) and mechanical loading on chondrogenesis in vitro

**DOI:** 10.1038/s41598-021-99614-0

**Published:** 2021-10-12

**Authors:** N. Pötter, F. Westbrock, S. Grad, M. Alini, M. J. Stoddart, H. Schmal, D. Kubosch, G. Salzmann, E. J. Kubosch

**Affiliations:** 1 AO Research Institute Davos, Davos, Switzerland; 2 Department of Orthopedics and Trauma Surgery, Albert-Ludwigs University Medical Center Freiburg, Freiburg, Germany; 3 Schulthess Klinik, Zürich, Switzerland; 4 Department of Orthopaedic Surgery, University Hospital Odense, Odense, Denmark

**Keywords:** Medical research, Experimental models of disease

## Abstract

The aim of this work is to investigate the capability of PRP as an adjuvant therapy to autologous chondrocyte implantation (ACI) in combination with multi-axial load with respect to cartilage regeneration. Articular cartilage shows poor repair capacity and therapies for cartilage defects are still lacking. Well-established operative treatments include ACI, and growing evidence shows the beneficial effects of PRP. Platelets contain numerous growth factors, among them transforming growth factor beta (TGF-β). Dynamic mechanical loading is known to be essential for tissue formation, improving extracellular matrix (ECM) production. For our ACI model monolayer expanded human chondrocytes were seeded into polyurethane scaffolds and embedded in fibrin (hChondro), in PRP-Gel (PRP), or in fibrin with platelet lysate (PL), which was added to the media once a week with a concentration of 50 vol%. The groups were either exposed to static conditions or multi-axial forces in a ball-joint bioreactor for 1 h per day over 2 weeks, mimicking ACI under physiological load. The culture medium was collected and analyzed for glycosaminoglycan (GAG), nitrite and transforming growth factor beta 1 (TGF-β1) content. The cell-scaffold constructs were collected for DNA and GAG quantification; the expression of chondrogenic genes, TGF-β and related receptors, as well as inflammatory genes, were analyzed using qPCR. Loading conditions showed superior chondrogenic differentiation (upregulation of COL2A1, ACAN, COMP and PRG4 expression) than static conditions. PRP and PL groups combined with mechanical loading showed upregulation of COL2A1, ACAN and COMP. The highest amount of total TGF-β1 was quantified in the PL group. Latent TGF-β1 was activated in all loaded groups, while the highest amount was found in the PL group. Load increased TGFBR1/TGFBR2 mRNA ratio, with further increases in response to supplements. In general, loading increased nitrite release into the media. However, over time, the media nitrite content was lower in the PL group compared to the control group. Based on these experiments, we conclude that chondrogenic differentiation is strongest when simulated ACI is performed in combination with dynamic mechanical loading and PRP-gel or PL supplementation. An inflammatory reaction was reduced by PRP and PL, which could be one of the major therapeutic effects. Loading presumably can enhance the action of TGF-β1, which was predominantly activated in loaded PL groups. The combination of load and PRP represents an effective and promising synergy concerning chondrocyte-based cartilage repair.

## Introduction

Young individuals with a high level of activity have an increased risk of suffering from sports injuries associated with cartilage damage^1^. Subsequent inadequate loading and metabolic changes within the joint lead to further cartilage loss^2,3^. Knee injuries are associated with a high risk of developing post-traumatic osteoarthritis (PTOA)^2,4^. Early intervention is desirable to prevent young patients from developing early OA. A surgical treatment to re-establish load-bearing tissue capacity is (matrix-associated) autologous chondrocyte implantation (ACI), which has shown excellent clinical outcome^5–9^. Joint regeneration and homeostasis also depend on a balance of signaling molecules which ensures tissue integrity and function^10,11^.

Both trauma and the following surgical intervention constitute changes in homeostasis. In particular, processes such as inflammation and catabolism should be taken into account when choosing the best available therapy. Recently, a substance with a possible disease-modifying and chondroprotective effect in OA, platelet-rich plasma (PRP), is receiving increasing attention. Besides excellent clinical improvement for patients suffering from a degenerative joint disease^12–16^, PRP has shown beneficial effects on proteoglycan and collagen synthesis^17,18^, cell proliferation^17^ and redifferentiation in vitro^18^. Furthermore, it showed a stimulating effect on the synthesis of proteoglycan 4 and hyaluronic acid, which provide lubrication of the joint surfaces^19,20^. Platelets contain numerous growth factors (e.g. TGF-β, PDGF, VEGF, FGF, HGF, EGF and IGF) plausibly inducing the described effects. TGF-β, PDGF, FGF and IGF-1 promote cell proliferation. TGF-β, PDGF and IGF regulate the ECM synthesis. The functions of VEGF, HGF and FGF-2 include stimulation of angiogenesis. Therefore, PRP activates a variability of favorable but also possibly detrimental signaling cascades^21–23^. Among the growth factors, transforming growth factor beta 1 (TGF-β1) is one of the most interesting regarding cartilage tissue regeneration. It is known for its role in anabolic and regenerative processes, proliferation and modulation of anti-inflammatory processes^24–26^. TGF-β1 is an elementary part of signaling pathways for cartilage maintenance^27^ and plays an important role in tissue engineering approaches. It is known that a mechanical stimulus can activate TGF-β1^28–30^. As a consequence, physiological load seems to be an important determinant for the bioavailability of active TGF-β1.

This study aimed to investigate PRP as an adjuvant therapy to ACI in coaction with multi-axial load in cartilage regeneration. In particular, we wanted to evaluate whether the chondroprotective and cartilage growth-promoting pathway (TGFBR1/TGFBR2 profile) can be modified in chondrocytes using the combination of multi-axial load and PRP/PL.

We hypothesized that PRP/PL in combination with multi-axial load might have favorable effects on chondrocytes and biochemical cytokine composition.

## Methods

**Figure 1:**
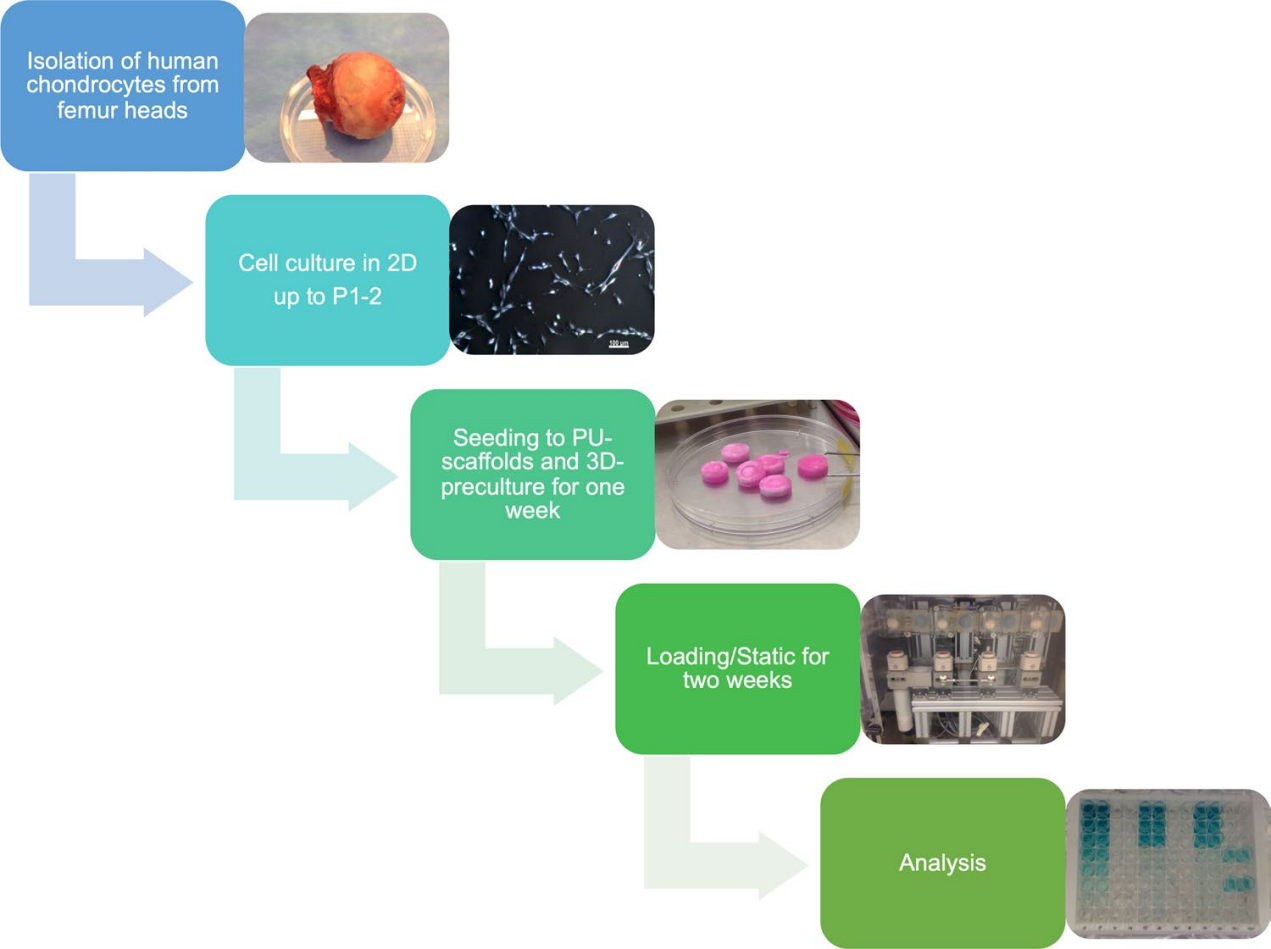
Experimental design.

Chondrocytes were isolated from femoral heads of four independent human donors, which were removed during arthroplasty operation at Davos Hospital, with ethical approval from the ethic commission “Kantonale Ethikkommission Zürich” (KEK-ZH-NR: 2010-0444/0).The chondrocytes were isolated on the day the femoral head was obtained (Experimental Design in Fig. 1). All experiments and analyses were done in 3–4 technical replicates and the experiment was repeated four times (Table 1).

**Table 1:** Information on each specific donor.

Donor no	Age	Sex	Color
#1	69	m	Black
#2	49	f	Blue
#3	81	f	Red
#4	82	f	Green

The color column defines each data point in Figs. 4, 5 and 6.

### Cell isolation and expansion

Cartilage was harvested from unscathed parts of the joint and cut into 9–25 mm^2^ pieces. After washing in PBS, the tissue was predigested in 0.1% Pronase for 105 min, followed by a second washing step. Next, the samples were digested for 14 h in 600 U/ml collagenase 2 in a spinner flask at 37 °C, 5% CO_2_ and 95% humidity. After filtration through a 40 µm cell strainer, the cells were suspended in High Glucose Dulbecco’s Modified Eagle Medium (DMEM-HG) with 10% Fetal Calf Serum (FCS). To attain a sufficient cell number, the chondrocytes were seeded in T300 flasks at a density of 3000–4000 cells/cm^2^ and expanded in monolayer up to passage 2 in DMEM-HG, substituted with 1% HEPES Buffer, 1% Penicillin/Streptomycin, 1% Glutamine, 10% FCS, 1 ng/ml TGF-β1 and 5 ng/ml bFGF-2.

### PRP preparation

Leukocyte-poor PRP was produced from human thrombocyte concentrates obtained from the blood bank Chur, Switzerland. Platelet concentrates of 10 Rhesus positive donors with blood type 0 were pooled and contained an average of 765,500 thrombocytes/µl. To obtain PRP with a tenfold concentration of the physiological blood concentration (140,000–345,000/µl), the concentrates were centrifuged at 2000*g* for 15 min, and the pellet was resuspended in half of the subtracted volume (Classification: PAW P4, exogenous activation, B; Mishra Type 4A; WBC < 20/µl). To obtain platelet lysate (PL) with a fivefold increase of baseline, the thrombocyte concentrate was sonicated for 15 min to activate the platelets (Classification: PAW P3, exogenous activation, B; Mishra Type 4A; WBC < 10/µl). Samples were aliquoted and frozen at − 20 °C. After thawing, PL was centrifuged at 18,000*g* for two minutes to remove cell debris.

### Seeding to scaffolds

To mimic an ACI implant under load in controlled laboratory conditions, cells were seeded into a polyurethane-gel matrix to provide a resilient three-dimensional matrix. Polyurethane(PU)-scaffolds (8 × 4 mm) were produced with a pore size of 150–300 µm^31^. 5 × 10^6^ cells were embedded into a fibrin or PRP gel and dispensed to the PU-scaffolds. Fibrinogen (Baxter) and PRP were activated with a 0.5 U/ml thrombin solution (Baxter).

All cell-scaffold constructs were kept in custom-made holders and the culture medium was changed every two to three days. For adaption to a three-dimensional culture, all groups were left in free swelling condition for one week. The medium contained DMEM-HG 1% Penicillin/Streptomycin, 1% Insulin–transferrin–sodium selenite + 1 (ITS + 1), 1% non-essential amino acids, 1% L-ascorbic-acid-2-phosphate (50 µg/ml), 0.1% 6-Aminocaproic Acid (EACA) (5 µM).

Groups were divided into:

- Static
  1. hChondro (fibrin)
  2. PRP-gel
  3. PL (fibrin-gel with PL added once a week with a concentration of 50 vol%)
- Loading
  4. hChondro (fibrin)
  5. PRP-gel
  6. PL (fibrin-gel with PL added once a week with a concentration of 50 vol%)

### Mechanical stimulation

In a ball-joint bioreactor^32^ ACI implants were exposed to complex loading for one hour per day, five times a week, over two weeks at 37 °C, 5% CO_2_ and 95% humidity. Using a ceramic hip ball which was moved on a vertical axis, first, a preload of 10% of the scaffold height (0.4 mm) was applied. The multi-axial load consisted of a ± 0.2 mm dynamic compression and shear forces induced by ball rotation of ± 25° at 1 Hz. Load sensors monitored the transmission of forces on the PU-scaffold.

### Analysis

The medium was frozen at every medium change and a small sample volume of 150 µl was taken directly after mechanical stimulation to analyze for active metabolites. At the experiment endpoint, cell-scaffold constructs were cut into halves.

### Biochemical assays (GAG, DNA, NO)

Cell-scaffold samples were digested for 16 h at 56 °C using proteinase K (0.5 mg/ml).

Glycosaminoglycans (GAG) were measured from constructs and collected medium at days 3, 5, 8, 10, 12, 15, 17 and 19 using a 1,9-dimethyl-methylene blue (DMMB)-assay (Sigma-Aldrich). A standard curve was prepared using bovine chondroitin sulfate and measured in a Victor 3 microplate reader at 535 nm.

DNA content was measured using a Hoechst 33258 fluorescence assay. A standard curve was prepared with calf thymus dsDNA and analyzed using the same microplate reader at 360 nm (excitation) and 465 nm (emission).

As a progression parameter for inflammatory reaction, nitrite in media was measured. Griess reagent was used according to the manufacturer’s instructions.

### RNA extraction, reverse transcription, qPCR

Cell-scaffold samples were homogenized with a TissueLyser (Qiagen) in TRI® Reagent at 25 Hz for 3 min. Samples were centrifuged at 12,000*g* for 10 min and 0.1 ml 1-Bromo-3-chloropropane was added per 1 ml TRI. RNA from the aqueous phase was precipitated by adding isopropanol. After centrifugation, the RNA pellet was washed with 75% ethanol and resuspended in RNase-free water. The cDNA was synthesized using TaqMan reverse transcription reagents and 1 µg total RNA. qPCR was realized using a QuantStudio™ 6 Flex Real-Time PCR System. RPLP0 was used as housekeeping-gene and relative gene expression was calculated compared to day 0, using the 2^−ΔΔCT^ method.

### ELISA

Medium samples of 150 µl were taken directly after mechanical stimulation using protein low binding tips to measure total and active TGF-β. The ELISA was performed according to the manufacturer's protocol (R&D Systems).

### Statistics

All statistical analyses were performed using GraphPad Prism 7.0. Results of technical replicates were averaged, and the respective mean was used for statistical analysis. Groups were tested for normality using the Shapiro–Wilk test. Two way ANOVA was performed for parametric data; nonparametric data was analyzed with the Kruskal–Wallis test. # defines significance between static and loaded, * defines significance between specific groups. One symbol = *p* < 0.05, two symbols = *p* < 0.01, three symbols = *p* < 0.001.

### Ethical approval

Kantonale Ethikkommission Zürich (KEK-ZH-NR: 2010-0444/0). All methods were carried out in accordance with relevant guidelines and regulations.

### Informed consent

Informed consent was obtained from all individual participants of the study.

## Results

### Biochemical assays (GAG, NO)

**Figure 2:**
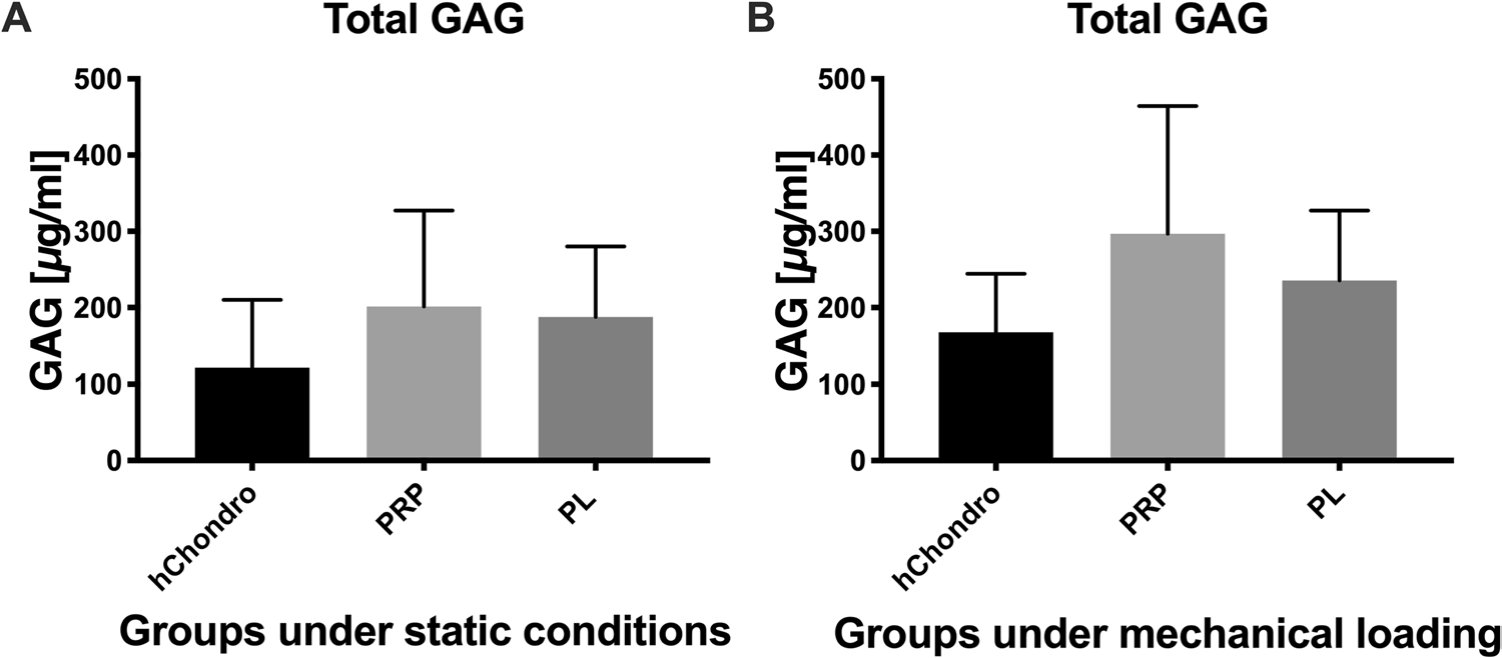
GAG quantification of human chondrocytes (hChondro), PRP and PL groups shown under static conditions (**A**) and mechanical stimulation (**B**), shown as mean + SD, statistical significance was defined as *p* < 0.05.

Total GAG (GAG in the construct + cumulative GAG in the medium) was higher but not significant in PRP and PL groups compared to hChondro groups exposed to loading (PRP: 296.76 ± 167.51 µg/ml; PL: 235.76 ± 91.64 µg/ml; hChondro: 167.78 ± 76.70 µg/ml) and static conditions (PRP: 201.46 ± 125.65 µg/ml; PL: 187.74 ± 92.65 µg/ml; hChondro: 121.92 ± 88.16 µg/ml) (Fig. 2).

**Figure 3:**
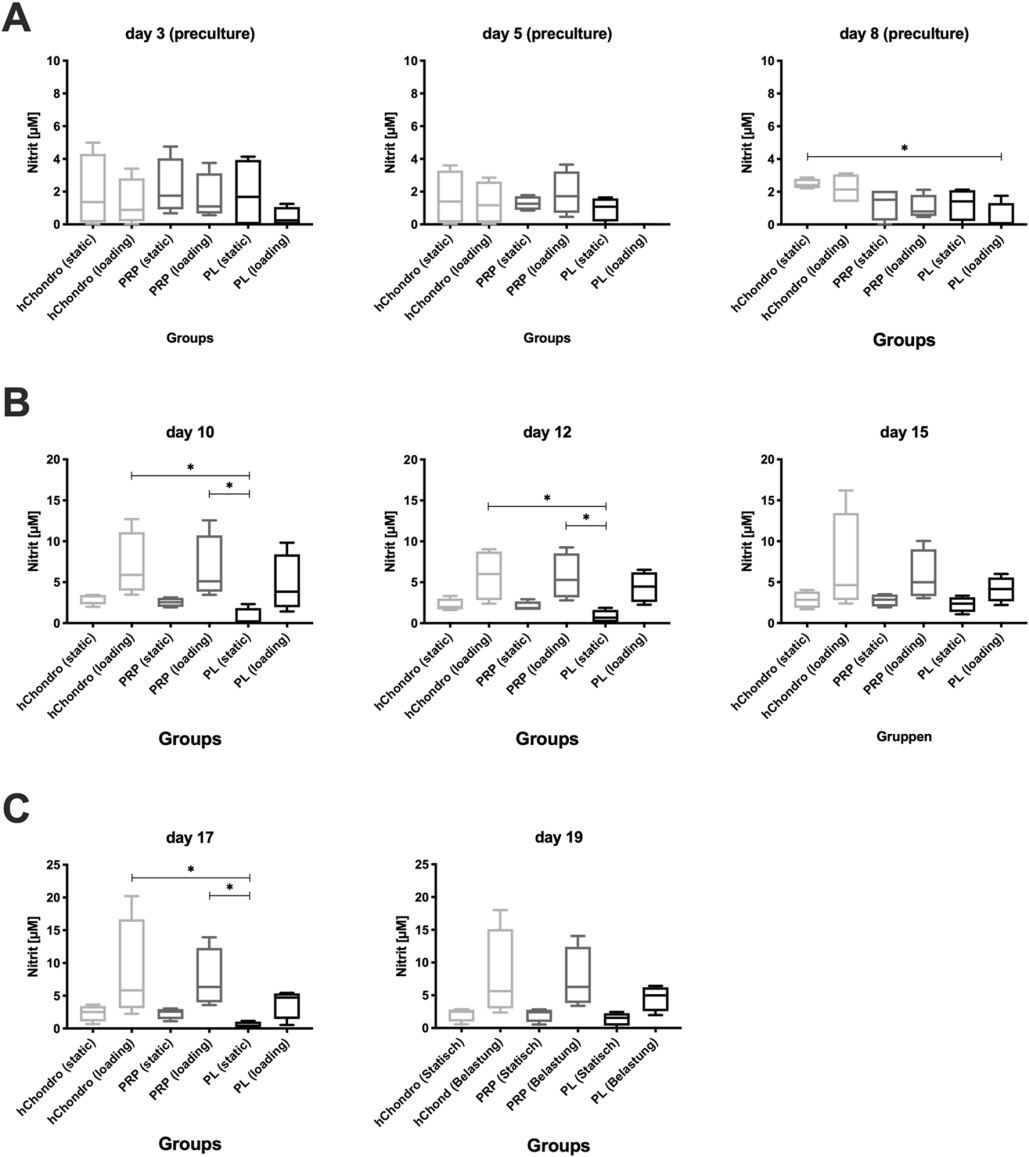
Quantitative measurement of nitrite over time under preculture (**A**), mechanical loading during the 1st week (**B**) and the 2nd week (**C**), * defines significance between specific groups *p* < 0.05.

Nitrite was measured over time as an indirect marker for NO production (Fig. 3)^33^. Under static conditions (Fig. 2A–C) nitrite was generally lower and reduced over time. An increase of nitrite was seen with the beginning of loading (Fig. 2B–C). This increase was mitigated when load was combined with PL.

### qPCR

QPCR examination showed large interindividual differences in gene expression responses. Due to this heterogeneity, significance often could not be displayed, despite clear trends being visible.

**Figure 4:**
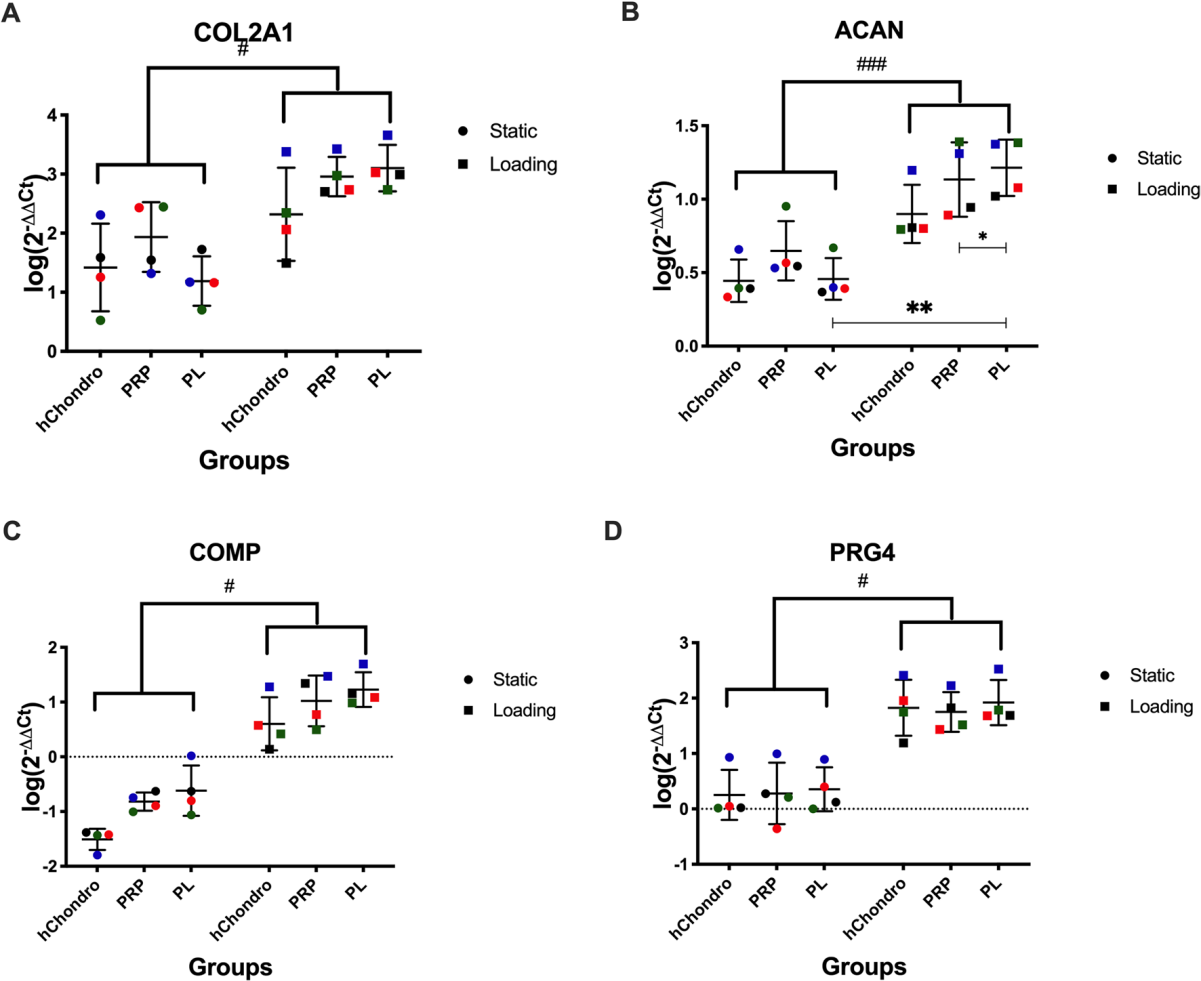
Influence of PRP-Gel, PL supplementation and multi-axial loading on mRNA levels of COL2A1 (**A**), ACAN (**B**), COMP (**C**), PRG4 (**D**). Relative gene expression (log(2^−ΔΔCT^)) was transformed by log(10) and visualized in scatter dot plots with different colors for each donor and lines at mean ± SD, # defines significance between static and loaded, * defines significance between specific groups. One symbol = *p* < 0.05, two symbols = *p* < 0.01, three symbols = *p* < 0.001.

All chondrogenic differentiation markers showed a strong and often significant upregulation of gene expression after loading (Fig. 4). The highest expression of COL2A1, ACAN and COMP was seen in the loaded PL group. In COL2A1 (Fig. 4A) a 100–1000-fold change in the loaded PL group compared to hChondro static could be observed. COMP appeared to have the greatest response to supplements, but the trends did not reach significance (Fig. 4C). A synergistic effect of mechanical stimulation and PL can be presumed. PRG4 (Fig. 4D) was upregulated in all groups exposed to load.

**Figure 5:**
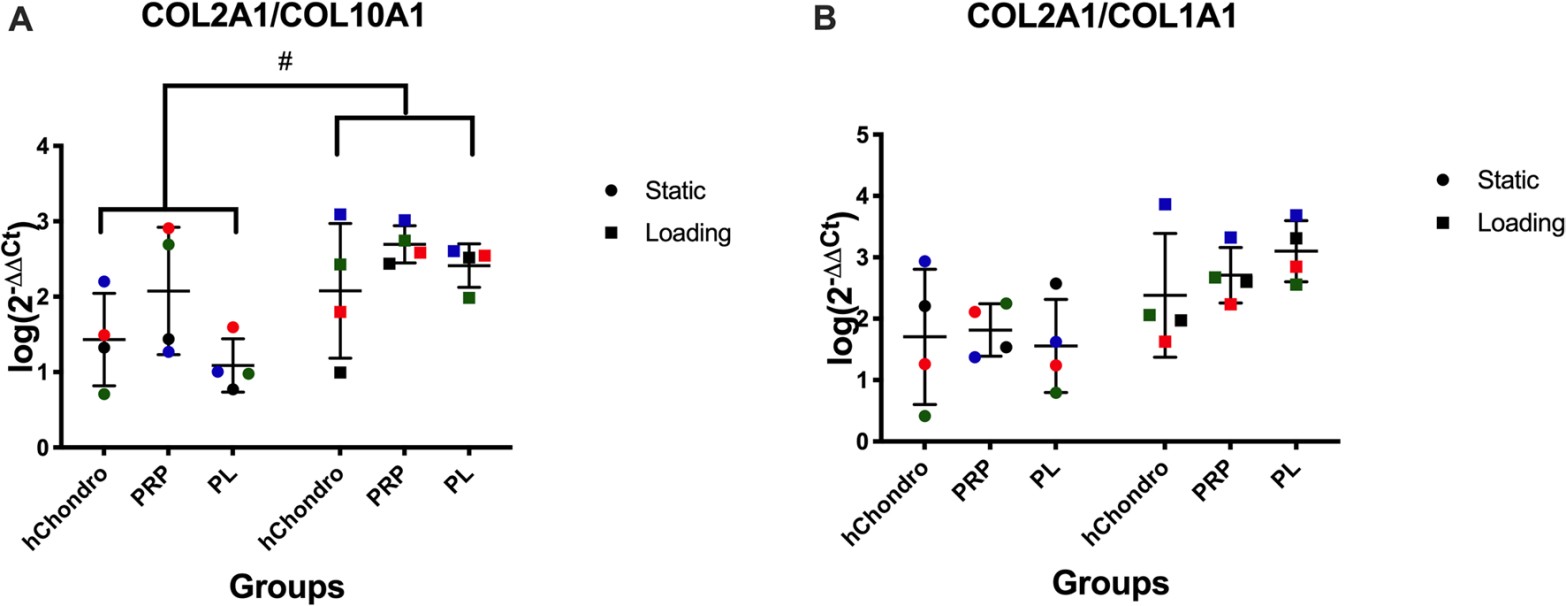
Influence of PRP-Gel, PL supplementation and multi-axial loading on mRNA levels of COL2A1/COL10A1 ratio (**A**) and COL2A1/COL1A1 ratio (**B**). Relative gene expression (log(2^−ΔΔCT^)) was transformed by log(10) and visualized in scatter dot plots with different colors for each donor and lines at mean ± SD, statistical significance was defined as ^#^*p* < 0.05.

COL2A1/COL10A1 (Fig. 5A) and COL2A1/COL1A1 ratios (Fig. 5B) were calculated to assess chondrocyte hypertrophy and dedifferentiation, respectively. An increased COL2A1/COL10A1 ratio was observed in loaded groups.

**Figure 6:**
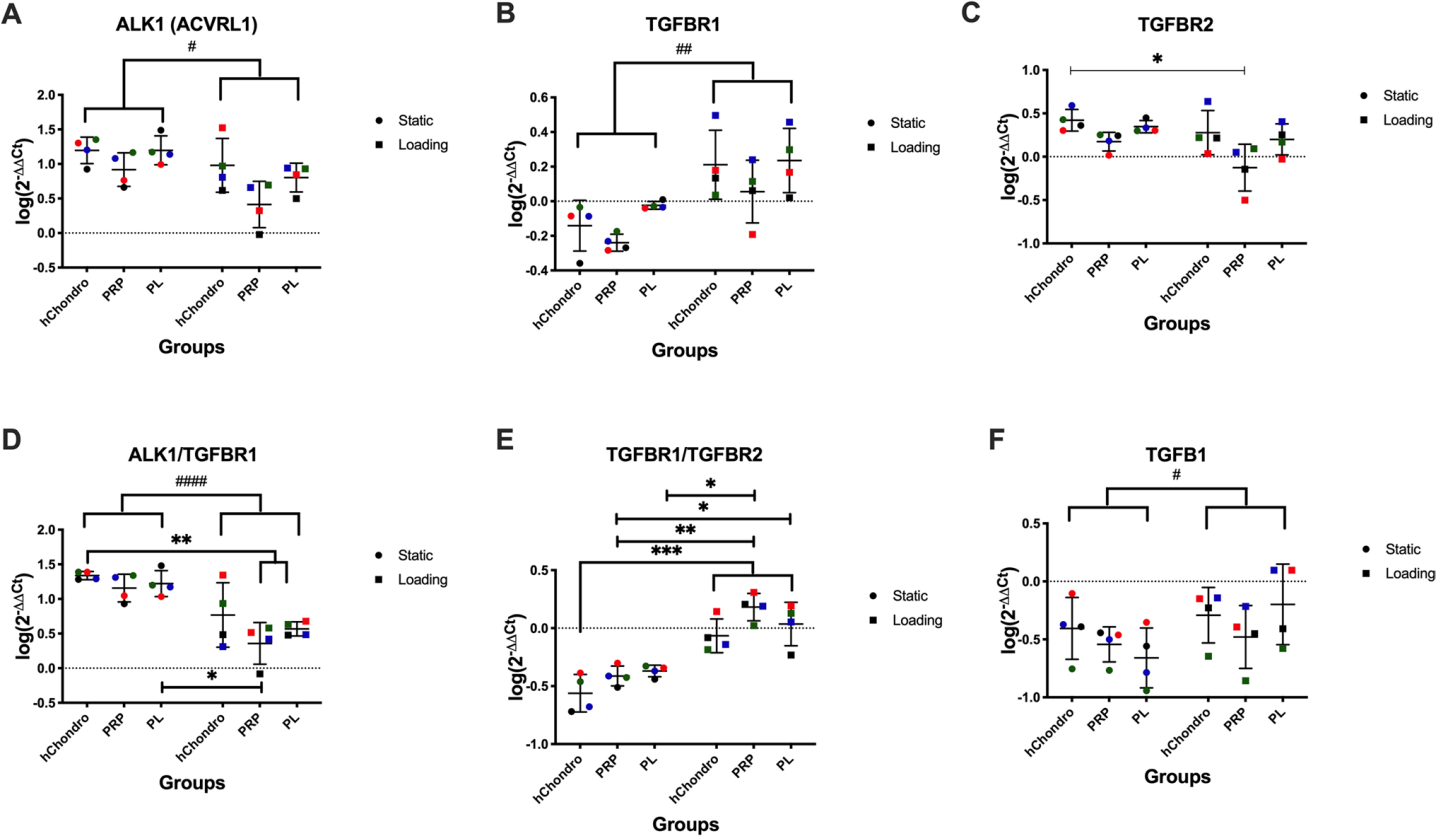
Influence of PRP-Gel, PL supplementation and multi-axial loading on mRNA levels of ALK1 (**A**), TGFBR1 (**B**), TGFBR2 (**C**), ALK1/TGFBR1 ratio (**D**) TGFBR1/TGFBR2 ratio (**E**) and TGF-β1 (**F**). Relative gene expression (log(2^−ΔΔCT^)) was transformed by log(10) and visualized in scatter dot plots with different colors for each donor and lines at mean ± SD, # defines significance between static and loaded, * defines significance between specific groups. One symbol = *p* < 0.05, two symbols = *p* < 0.01, three symbols = *p* < 0.001.

ALK1 (Fig. 6A) showed a decreased expression with load, TGF-β receptor 1 (TGFBR1) showed an increased expression with load (Fig. 6B), while TGFBR2 was largely unaffected (Fig. 6C) . The ratio of ALK1 and TGFBR2 against TGFBR1 was calculated to estimate a relation of activated signaling cascades. ALK1/ TGFBR1 ratio significantly decreased under complex load, particularly in loaded PRP and PL groups, suggesting a shift towards TGFBR1 signaling (Fig. 6D). The TGFBR1/TGFBR2 increased with load and with supplementation (Fig. 6E), suggesting a shift towards Smad 2/3 signaling. In contrast, TGFB1 expression did only change with loading (Fig. 6F).

#### TGF-β1-enzyme-linked immunosorbent assay (ELISA)

**Figure 7:**
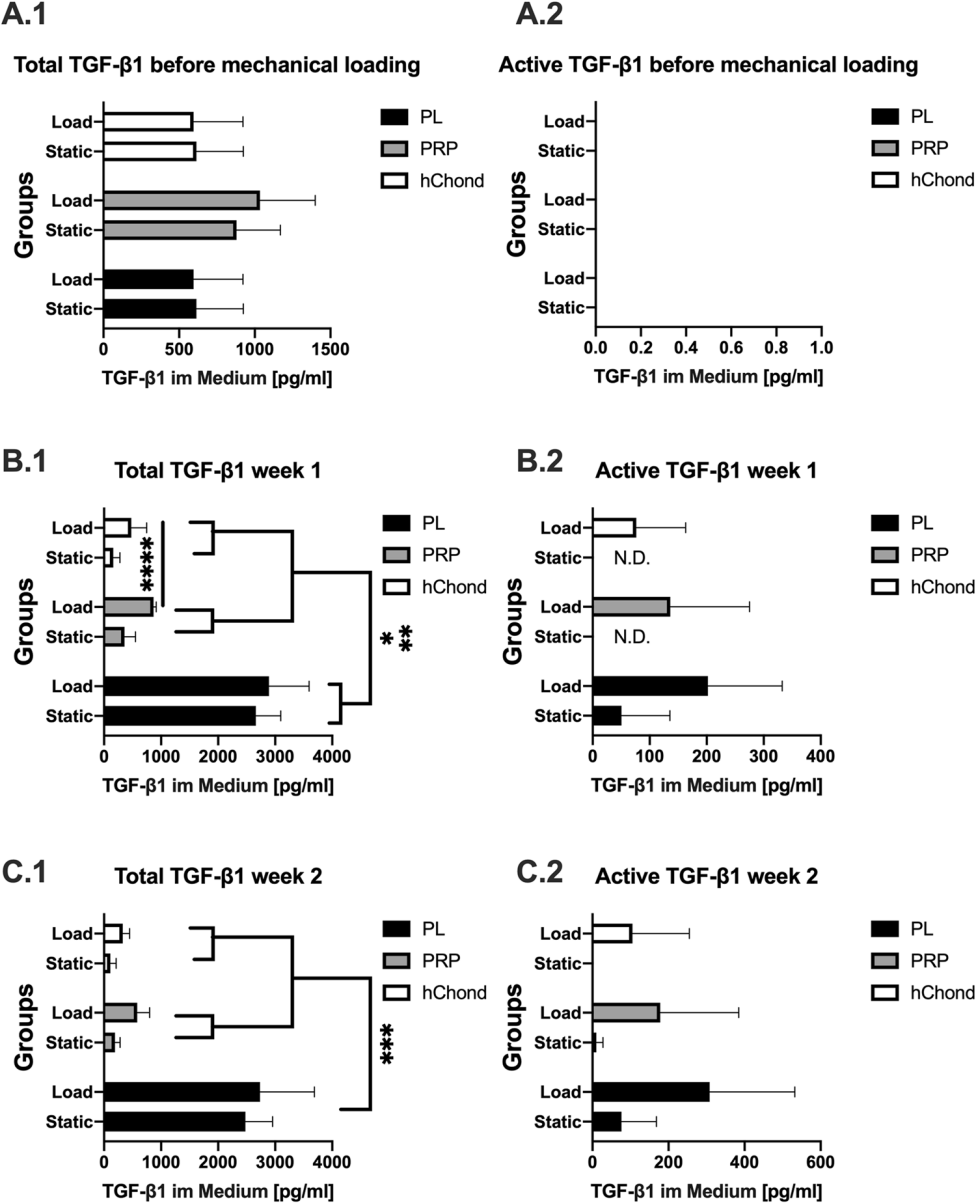
Total and activated TGF-β1 in cell culture medium, structured in time by (**A**) preculture before mechanical loading, (**B**) week 1 and (**C**) week 2. Data is shown as mean ± SD, statistical significance was defined as *p < 0.05, **p < 0.01, ***p < 0.001, ****p < 0.0001.

For an accurate assessment of TGF-β1 levels, active (A-C.2) and total (active and latent) (A-C.1) amounts were determined from pooled media samples of each week (Fig. 7). Striking was the amount of TGF-β1 measured in PL groups for week 1 (2661.40 ± 435.36 and 2891.44 ± 703.79 pg/ml) (B.1) and week 2 (2478.93 ± 476.26 and 2736.03 ± 953.70 pg/ml) (C.1). Both PL groups showed significantly higher activated TGF-β1 concentration than the control group (week 1 p = 0.0466 and week 2 p = 0.0370) (161.65 ± 116.89 in week 1 and 112.53 ± 97.51 pg/ml in week 2).

Looking at the active TGF-β1 (A-C.2), no active TGF-β1 could be measured before the onset of mechanical stimulation. The highest concentration of active TGF-β1 was measured in the loaded PL group, showing an increase over time (week 1: 202.29 ± 130.33; week 2: 308.32 ± 223.94 pg/ml).

## Discussion

The main finding of this study was that PRP-Gels and supplementation of PL could positively influence cartilage related markers. PRP/PL in combination with multi-axial load seem to have favorable effects on chondrocytes and biochemical cytokine composition. We could validate the anabolic effect of PRP on chondrocytes^17,18^. GAG synthesis was higher in PRP- and PL-complemented groups. Gene expression of COL2A1, ACAN and COMP was upregulated by these supplements in combination with mechanical stimulation.

PL supplementation without mechanical loading did not show significantly increased gene expression for COL2A1, ACAN and PRG4 compared to the control group. Therefore a combination of load and supplementation seems to be beneficial or even necessary for improved chondrogenesis. This confirms previous studies, which could show an increased formation of ECM proteins both through PRP^17,18^ and loading^31,34^. Interestingly, both effects seem to be synergistic.

With the onset of mechanical stimulation, a strong increase of nitrite in the medium could be seen. If one considers nitrite as an indicator for inflammatory processes, mechanical load seems to have a pro-inflammatory effect. This effect could already be shown in other experiments with this bioreactor^28^. Interestingly, nitrite concentration over time was significantly lower in PL groups compared to control groups. Assuming that mechanical stimulation is essential for forming an adequate cartilage implant and striving for a post-operative rehabilitation protocol that allows mobility for the patient’s daily activities, an increased inflammation has to be managed. In this regard, adjuvant injection of PRP or PL can potentially provide great benefit and reduce the extent of inflammation.

In cartilage tissue, TGF-β1 is involved in important regulatory processes. With the addition of PRP or PL, we add exogenous TGF-β1, which can contribute to tissue preservation^35–37^. A few signaling pathways of TGF-β have been discovered: First, TGF-β1 binds TGF-β receptor 2 (TGFBR2), a kinase which can activate TGF-β receptor 1 (TGFBR1, ALK5 (*activin receptor-like kinase 1))*, stimulating further signaling cascades^38^. In cartilage, the TGFBR1/Smad2/3 signaling pathway seems to contribute to cartilage maintenance and stimulates the production of ECM proteins^35–37,39^. In addition, TGF-β1 receptor 2 can interact with ALK1, which in turn activates the Smad1/5/8 pathway^40,41^. This signaling can promote terminal differentiation in chondrocytes indicating typical changes of chondrocytes in OA development^42,43^. On the other hand, the Smad2/3 pathway seems to have an inhibiting effect on OA development^42^: A TGFBR1 knockout in mice leads to degradation of articular cartilage, synovial hyperplasia, formation of osteophytes, subchondral sclerosis, decreased expression of anabolic factors, increased expression of catabolic factors and increased chondrocyte apoptosis^37^. Thus, the same ligand can provoke contrary effects, depending on the susceptibility of the tissue and the proportion of the different receptors for the ligand^40^. The response of cells to TGF-β1 depends on the activated signaling cascade: Via the TGFBR1/Smad2/3 pathway, expression of important ECM proteins, for example, PRG4 and aggrecan, is induced^37,39^. In contrast, ALK1 leads via Smad1/5/8 to terminal differentiation and advances OA-associated processes, e.g., osteophyte formation and increased expression of MMP-13^39,42,43^. In aging tissue or with the progression of OA, a change in TGF-β1 signaling with reduction of TGF receptor 1 was observed^39,41,44^.

Therefore, added TGF-β could potentially have harmful effects. To evaluate the balance of TGF-β receptors, the ALK1/TGFBR1 ratio was established by van der Kraan et al. An increase of this ratio could be seen during the development of OA^39^. We could observe a strong reduction of the ALK1/TGFBR1 ratio in PRP and PL groups which were exposed to dynamic compression and shear forces suggestive of a beneficial effect. This shift could indicate that activated TGF-β1 has a beneficial effect regarding anabolic processes and improved tissue regeneration.

Interestingly, we did not observe an increase of TGF-ß1 at the endpoint of our experiment at week 3. In contrast to our results, Madej et al. could observe an upregulation of the TGF-β1 gene directly at 2 and 6 h after loading^41,45^. Differences in our study include a longer time frame and the different time points at which we monitored the gene expression of the cells. More time points were not feasible due to the number of cells required for this experimental design. Moreover, post-transcriptional gene silencing can affect translation and, therefore, actual protein concentration^46,47^. Yet, the data on protein level suggests that mechanical loading enhanced the synthesis of TGF-β1.

Results from TGF-β1 ELISA allow us to distinguish between total and active TGF-β1. Active TGF-β1 is the relevant protein for signaling because only the active form can bind its receptor. In the first week, we could not measure any active TGF-β1. It is possible that added TGF-β undergoes fast usage in the first week. With the onset of mechanical stimulation, activated TGF-β1 could be measured, which corresponds to previously published work^28^. Mechanical stimulation can release and even activate TGF-β1 and therefore seems pivotal for the provision of active TGF-β1 protein. In the loaded PL group, the largest amount of active TGF-β1 was available. This occurred mainly in the loaded PL groups. The activated TGF-β1 can then signal via a modified ALK1/TGFBR1 ratio. Again, this could provide for a beneficial stimulus and a less catabolic environment. After ACI, differentiated rehabilitation protocols together with PL injections could improve transplant quality. Keeping a close check on the clinical outcome, including follow-up injections, appears to be worthwhile.

A limitation of our study is that we did not analyze other growth factors (like PDGF, VEGF, FGF, HGF, EGF and IGF), which potentially affect tissue homeostasis. Also, the effects of other molecules from the released granules were not evaluated in our study. E.g., arachidonic acid, released by platelets and in interaction with neutrophils, can be converted to lipoxin, a potent anti-inflammatory protein that might have an important impact on the progression of the healing cascades^23^.

We focused on one protein from the TGF-β family. There are more than 29 ligands for the TGF-β-receptors^40^. An influence of other proteins contained in PRP (such as BMP2, EGF, IGF, FGF and others) cannot be excluded in the present model.

We are aware that our study has a small sample number of chondrocyte donors (n = 4), and polyurethane (PU) is not commonly used for ACI in vivo. Yet, in the ACI model we used, PU is well evaluated and we reduced the interdonor-variation of PRP using pooled PRP (10 donors).

Further studies are necessary to determine the effect of PRP and PL on TGF-β signaling, and their involvement in inflammation pathways. The use of leukocyte-rich compared to leukocyte-poor PRP should be further evaluated as leukocyte-rich PRP has also shown excellent results for full-thickness cartilage defects and might involve other anti-inflammatory pathways^48^. Subsequent in vitro studies could involve different types of PRP and co-cultures with other cell types from the joint, for example, synoviocytes.

## Conclusion

PRP or PL seem to be a supporting adjuvant for cartilage *tissue engineering* products under loading conditions. PRP or PL supplementation together with mechanical loading show synergistic effects while at the same time suppressing inflammation. Our study showed positive effects on chondrogenesis, reduction of the inflammatory response and an improved effect of TGF-β1 due to a change in the TGF-β receptor balance. With regard to inflammatory conditions after injury or surgical intervention, PRP could inhibit or reduce inflammatory influences and prevent the development of PTOA. Adapted rehabilitation protocols for optimized mechanostimulation could even enhance these positive effects.

A possible clinical application could involve both a PRP/PL usage in vitro during the manufacturing process of the implant, as well as *per injectionem *in vivo: PRP can support hyaline-like tissue formation in the three-dimensional phase of the ACI product in vitro and promote further directed differentiation of chondrocytes postoperatively in vivo.

## References

[1] Riordan EA, Little C, Hunter D (2014). Pathogenesis of post-traumatic OA with a view to intervention. Best Pract. Res. Clin. Rheumatol..

[2] Kramer WC, Hendricks KJ, Wang J (2011). Pathogenetic mechanisms of posttraumatic osteoarthritis: Opportunities for early intervention. Int. J. Clin. Exp. Med..

[3] Southworth TM, Naveen NB, Nwachukwu BU, Cole BJ, Frank RM (2019). Orthobiologics for focal articular cartilage defects. Clin. Sports Med..

[4] Lohmander LS, Englund PM, Dahl LL, Roos EM (2007). The long-term consequence of anterior cruciate ligament and meniscus injuries: Osteoarthritis. Am. J. Sports Med..

[5] Crawford DC, Heveran CM, Cannon WD, Foo LF, Potter HG (2009). An autologous cartilage tissue implant neocart for treatment of grade III chondral injury to the distal femur: Prospective clinical safety trial at 2 years. Am. J. Sports Med..

[6] Kon E (2011). Second-generation autologous chondrocyte implantation: Results in patients older than 40 years. Am. J. Sports Med..

[7] Mistry H (2017). Autologous chondrocyte implantation in the knee: Systematic review and economic evaluation. Health Technol. Assess. (Rockv)..

[8] Ogura T, Bryant T, Minas T (2017). Long-term outcomes of autologous chondrocyte implantation in adolescent patients. Am. J. Sports Med..

[9] Ogura T, Bryant T, Mosier BA, Minas T (2018). Autologous chondrocyte implantation for bipolar chondral lesions in the tibiofemoral compartment. Am. J. Sports Med..

[10] Lories RJU (2008). Joint homeostasis, restoration, and remodeling in osteoarthritis. Best Pract. Res. Clin. Rheumatol..

[11] Kubosch EJ (2016). Clinical outcome and T2 assessment following autologous matrix-induced chondrogenesis in osteochondral lesions of the talus. Int. Orthop..

[12] Sánchez M, Anitua E, Azofra J, Aguirre JJ, Andia I (2008). Intra-articular injection of an autologous preparation rich in growth factors for the treatment of knee OA: A retrospective cohort study. Clin. Exp. Rheumatol..

[13] Marmotti A (2015). PRP and articular cartilage: A clinical update. Biomed. Res. Int..

[14] Kon E (2010). Platelet-rich plasma: Intra-articular knee injections produced favorable results on degenerative cartilage lesions. Knee Surg. Sport. Traumatol. Arthrosc..

[15] Spaková T, Rosocha J, Lacko M, Harvanová D, Gharaibeh A (2012). Treatment of knee joint osteoarthritis with autologous platelet-rich plasma in comparison with hyaluronic acid. Am. J. Phys. Med. Rehabil..

[16] Gobbi A, Lad D, Karnatzikos G (2014). The effects of repeated intra-articular PRP injections on clinical outcomes of early osteoarthritis of the knee. Knee Surg. Sport. Traumatol. Arthrosc..

[17] Akeda K (2006). Platelet-rich plasma stimulates porcine articular chondrocyte proliferation and matrix biosynthesis. Osteoarthr. Cartil..

[18] Spreafico A (2009). Biochemical investigation of the effects of human platelet releasates on human articular chondrocytes. J. Cell. Biochem..

[19] Sakata R (2015). Stimulation of the superficial zone protein and lubrication in the articular cartilage by human platelet-rich plasma. Am. J. Sports Med..

[20] Patel S, Dhillon MS (2018). Platelet-rich plasma in orthopedics: efficacy, evidence, and evolution of our understanding over 10 years. J. Postgrad. Med. Educ. Res..

[21] Ornetti P, Nourissat G, Berenbaum F, Sellam J, Rhumatologie D (2016). Does platelet-rich plasma have a role in the treatment of osteoarthritis ?. Joint Bone Spine.

[22] Gonzalez dos Santos R (2021). The regenerative mechanisms of platelet-rich plasma : A review. Cytokine.

[23] Parrish WR, Roides B (2017). Physiology of blood components in wound healing: An appreciation of cellular co-operativity in platelet rich plasma action. J. Exerc. Sports Orthop.

[24] Sakata R, Reddi AH (2016). Platelet-rich plasma modulates actions on articular cartilage lubrication and regeneration. Tissue Eng. Part B Rev..

[25] Roman-Blas JA, Stokes DG, Jimenez SA (2007). Modulation of TGF-β signaling by proinflammatory cytokines in articular chondrocytes. Osteoarthr. Cartil..

[26] Langenmair, E. R., Kubosch, E. J., Salzmann, G. M., Beck, S. & Schmal, H. Clinical trial and in vitro study for the role of cartilage and synovia in acute articular infection. *Mediators Inflamm.* **2015** (2015). 10.1155/2015/430324 PMC4657131 26640325

[27] Scharstuhl A (2002). Inhibition of endogenous TGF-β during experimental osteoarthritis prevents osteophyte formation and impairs cartilage repair. J. Immunol..

[28] Gardner O, Fahy N, Alini M, Stoddart MJ (2016). Joint mimicking mechanical load activates TGFβ1in fibrin-poly(ester-urethane) scaffolds seeded with mesenchymal stem cells. J. Tissue Eng. Regen. Med..

[29] Ahamed J (2008). In vitro and in vivo evidence for shear-induced activation of latent transforming growth factor-21. Blood.

[30] Behrendt P (2020). Articular joint-simulating mechanical load activates endogenous TGF-β in a highly cellularized bioadhesive hydrogel for cartilage repair. Am. J. Sports Med..

[31] Grad S, Kupcsik L, Gorna K, Gogolewski S, Alini M (2003). The use of biodegradable polyurethane scaffolds for cartilage tissue engineering: Potential and limitations. Biomaterials.

[32] Wimmer MA (2004). Tribology approach to the engineering and study of articular cartilage. Tissue Eng..

[33] Schulz K, Kerber S, Kelm M (1999). Reevaluation of the Griess method for determining NO/NO-2 in aqueous and protein-containing samples. Nitric Oxide - Biol. Chem..

[34] Grad S (2012). Sliding motion modulates stiffness and friction coefficient at the surface of tissue engineered cartilage. Osteoarthr. Cartil..

[35] Zhang M (2010). Smad3 prevents β-catenin degradation and facilitates β-catenin nuclear translocation in chondrocytes. J. Biol. Chem..

[36] Yang X (2001). TGF-β/Smad3 signals repress chondrocyte hypertrophic differentiation and are required for maintaining articular cartilage. J. Cell Biol..

[37] Wang Q (2017). Cartilage-specific deletion of Alk5 gene results in a progressive osteoarthritis-like phenotype in mice. Osteoarthr. Cartil..

[38] Wrana JL, Attisano L, Wieser R, Ventura F, Massagué J (1994). Mechanism of activation of the TGF-β receptor. Nature.

[39] Blaney Davidson EN (2009). Increase in ALK1/ALK5 ratio as a cause for elevated MMP-13 expression in osteoarthritis in humans and mice. J. Immunol..

[40] Derynck R, Zhang YE (2003). Smad-dependent and Smad-independent pathways in TGF-β family signalling. Nature.

[41] Madej W (2016). Ageing is associated with reduction of mechanically-induced activation of Smad2/3P signaling in articular cartilage. Osteoarthr. Cartil..

[42] Madej W, van Caam A, Blaney Davidson EN, van der Kraan PM, Buma P (2014). Physiological and excessive mechanical compression of articular cartilage activates Smad2/3P signaling. Osteoarthr. Cartil..

[43] van der Kraan PM, Blaney Davidson EN, Blom A, van der Berg WB (2009). TGF-beta signaling in chondrocyte terminal differentiation and osteoarthritis. Modulation and integration of signaling pathways through receptor-Smads. Osteoarthr. Cartil..

[44] Blaney Davidson EN, Scharstuhl A, Vitters EL, van der Kraan PM, van den Berg WB (2005). Reduced transforming growth factor-beta signaling in cartilage of old mice: role in impaired repair capacity. Arthritis Res. Ther..

[45] Madej W, van Caam A, Blaney Davidson E, Buma P, van der Kraan PM (2016). Unloading results in rapid loss of TGFβ signaling in articular cartilage: role of loading-induced TGFβ signaling in maintenance of articular chondrocyte phenotype?. Osteoarthr. Cartil..

[46] Fire A (1998). Potent and specific genetic interference by double-stranded RNA in Caenorhabditis elegans. Nature.

[47] Xu J, Zhang JL, Zhang WG (2018). Antisense RNA: the new favorite in genetic research. J. Zhejiang Univ. Sci. B.

[48] Kazemi D, Fakhrjou A (2015). Leukocyte and platelet rich plasma (L-PRP) versus leukocyte and platelet rich fibrin (L-PRF) for articular cartilage repair of the knee : a comparative evaluation in an animal model. Iran Red Crescent Med.

